# CRISPR/CasRx-mediated RNA knockdown targeting β-catenin and Ihh signaling alleviates osteoarthritis

**DOI:** 10.1016/j.gendis.2024.101468

**Published:** 2024-11-16

**Authors:** Xingyun Huang, Jiamin Yu, Shixue Gou, Hongyu Qin, William W. Lu, Zhen Li, Liping Tong, Di Chen

**Affiliations:** a Research Center for Computer-aided Drug Discovery, Shenzhen Institute of Advanced Technology, Chinese Academy of Sciences, Shenzhen, Guangdong 518055, China; b Faculty of Pharmaceutical Sciences, Shenzhen University of Advanced Technology, Shenzhen, Guangdong 518055, China; c University of Chinese Academy of Sciences, Chinese Academy of Sciences, Beijing 100049, China; d Guangzhou National Laboratory, Guangzhou International Bio Island, Guangzhou, Guangdong 510005, China; e Division of Spine Surgery, The First Affiliated Hospital of Guangxi Medical University, Nanning, Guangxi 530021, China; f AO Research Institute Davos, Davos 7270, Switzerland

**Keywords:** CRISPR/CasRx, Ctnnb1, Indian hedgehog, Osteoarthritis, Smoothened

## Abstract

Osteoarthritis (OA) is a chronic degenerative joint disease. Currently, OA is incurable. Abnormal activation of canonical Wnt/β-catenin or Indian hedgehog (Ihh) signaling could lead to OA development and progression. This study aimed to determine if targeting β-catenin and Ihh signaling could yield an effective therapeutic intervention for OA disease. CRISPR/CasRx is a new RNA interference tool that can precisely and efficiently cleave single-strand RNAs. In this study, we screened CRISPR-derived RNA (crRNA) targeting *Ctnnb1* and *Smo in vitro* and selected two optimal crRNAs for each gene. CasRx-mediated *Ctnnb1* and *Smo* knockdown showed high efficiency and specificity with no obvious off-target effects *in vitro*. We then performed intra-articular injection of selected crRNAs driven by the adeno-associated virus into an OA mouse model. Micro-CT, histological, and histomorphometric analyses were conducted to evaluate the efficacy of CasRx approach on OA treatment. We found that the knockdown of *Ctnnb1* and *Smo* decelerated pathological damage in the keen joint of the experimental OA mouse model. Our findings suggest that CasRx-mediated *Ctnnb1* and *Smo* knockdown could be a potential strategy for OA treatment.

## Introduction

Osteoarthritis (OA) is a debilitating degenerative joint disease affecting large populations of patients worldwide that affects entire joint tissues, including the synovial tissue, articular cartilage, subchondral bone, and meniscus. Articular cartilage is the most affected tissue in the joint during OA progression.^1^, ^2^, ^3^ Currently, no effective drugs or other treatment procedures could cure OA disease or decelerate OA progression. Available treatment can only reduce the OA-associated pain and total joint replacement surgery is the strategy to deal with the end-stage OA disease.

To explore gene therapy of OA, we previously used the CRISPR/Cas9-mediated DNA editing approach to delete *IL-1β*, *Mmp13*, and *Ngf* in joint tissues. We found that deletion of these genes leads to a significant reversion of articular cartilage damage and pain induced by destabilization of medial meniscus (DMM) surgery, a well-established OA mouse model.^4^ These studies demonstrated the great potential of gene therapy for OA treatment. However, repair of DNA double-strand breaks caused by CRISPR/Cas9 could lead to large fragment deletions and chromosome rearrangements,^5^, ^6^, ^7^ so this approach has potential risks for permanent DNA modifications and limits the use of such technology, especially for the therapeutic and clinical usages.

In contrast to the DNA editing technique, gene therapy through RNA editing influences the targeted RNA only without affecting the DNA. In the past few years, RNA regulation has been used to treat OA and other rheumatic diseases in mice.^8^^,^^9^ RNA interference (RNAi) and microRNA (miRNA) reduce RNA expression and protect against arthritis diseases. However, extensive off-target transcript caused by traditional RNA editing techniques has always been the major concern.^10^

CRISPR/Cas13 is a new RNA interference tool, belonging to class 2 type VI CRISPR/Cas family,^12^ and it exerts higher specificity and efficiency than RNAi technique.^11^^,^^12^ Several members of the Cas13 family have been discovered, including Cas13a,^11^ Cas13b,^14^ Cas13d,^15^ CasX, and CasY.^13^ Among the various Cas13 subtypes, CasRx (RfxCas13d), belonging to the Cas13d subtype, shows higher specificity and higher activity than shRNA.^14^ Compared with other RNA editing techniques, the CasRx technique provides a much safer outcome for gene therapy.^15^ Adeno-associated virus (AAV) can easily package CasRx, due to its small size,^14^ and deliver it to the body. CasRx has been employed as a therapeutic tool in mouse models of autosomal-dominant hearing loss and osteoporosis.^16^^,^^17^ Catalytic active CasRx can be used for transcript knockdown, and dead CasRx (dCasRx) can be used to manipulate transcript splicing. A mis-splicing event was corrected by simultaneous targeting of multiple sites in the MAPT pre-mRNA through dCasRx in a dementia-related neuronal model derived from patients.^14^

*In vitro* and *in vivo* studies have shown that various growth factors and signaling molecules play important roles in the development of OA.^3^^,^^18^ Among them, there may be targets that CasRx can act on to treat OA. β-catenin is a central molecule in canonical Wnt signaling, encoded by *Ctnnb1*.^19^ Abnormal mechanical loading or mutations in Wnt decoy receptor *Frzb* cause onsite OA.^20^, ^21^, ^22^ *Frzb*-deficient mice showed OA-like phenotype.^23^ β-catenin up-regulation was detected in patients with OA or intervertebral disc degeneration.^24^^,^^25^ A mouse model with β-catenin specific activation in chondrocytes displayed an OA-like phenotype in the knee joint, temporomandibular joint, hip joint, and facet joint.^24^^,^^26^, ^27^, ^28^ These findings suggest that under pathological conditions, β-catenin activation is a crucial risk factor for OA development. The activation of Indian hedgehog (Ihh) signaling in chondrocytes leads to a severe OA-like phenotype in a genetic mouse model with Ihh signaling activation.^30^ Osteophyte formation and matrix mineralization are associated with abnormal hedgehog signaling activation in a temporomandibular joint OA mouse model.^29^ Smoothened (Smo) is a receptor necessary for the transmission of Ihh signaling, encoded by *Smo*.^30^ Knockdown of *Smo* by small interference RNA (siRNA) to inhibit the Ihh signaling pathway alleviates collagen-induced rheumatoid arthritis in mice.^31^

In this study, we focus on the Wnt/β-catenin and Ihh signaling pathways and explore the potential therapeutic application of CRISPR/Cas13d. We screened 20 CRISPR-derived RNAs (crRNAs) that match *Ctnnb1* or *Smo* transcripts, and selected two optimal crRNAs. Administration of CasRx could down-regulated the expression of the *Ctnnb1* and *Smo* transcripts without detectable off-target effects *in vitro*. We used AAV5 to deliver CasRx and crRNAs to the joint tissue of OA mice. Knockdown of *Ctnnb1* and *Smo* led to deceleration of OA progression. These results suggest that CasRx-mediated RNA editing is a potential therapeutic approach for OA treatment.

## Materials and methods

### Plasmid construction and AAV5 production

EF-1α-CasRx-T2A-GFP and U6-pre-crRNA backbone were given as a gift from the Lai Liangxue Laboratory. The crRNA fragments were inserted (Solution I, Takara) into the U6-pre-crRNA backbone after treatment with BbsI restriction enzyme (Thermo Fisher Scientific, USA). Vectors (ITR-U6-DR-crRNA-DR-crRNA-DR-EFS-NLS-CasRx-NLS-HA-pA-ITR) was constructed by Sangon Biotech (China). These vectors were used to generate AAV5 by PackGene Biotech (China). AAV5 (CMV-EGFP-WPRE-SV40pA) was purchased from PackGene Biotech (China). The titer of AAV5 was 1E+13 GC/mL. Vectors (ITR-U6-DR-crRNA-DR-crRNA-DR-EFS-NLS-CasRx-NLS-HA-T2A-GFP-pA-ITR) were produced by ligation of T2A-GFP fragments and linearized AAV plasmids (ClonExpress MultiS One Step Cloning Kit, Vazyme) after treatment with HindIII restriction enzyme (Thermo Fisher Scientific, USA). The primers used in this part are listed in Table S1.

### Cell culture and transfection

Fibroblast cell line NIH 3T3 (Procell, China) was cultured in DMEM (Gibco, USA) supplemented with 10% fetal bovine serum (Procell, China) and penicillin/streptomycin (Gibco, USA). All the cells were cultured in a humidified incubator at 37 °C with 5% CO_2_. When cells reached 85% confluence in 12-well plates, 1 μg U6-pre-crRNA and 2 μg CasRx-GFP (or 4 μg pre-crRNA-CasRx-GFP) plasmids were transfected into cells with Lipofectamine 3000 (Thermo Fisher Scientific, USA) in each well. 48 h after transfection, GFP-positive NIH 3T3 cells were sorted by BD FACSAria™ III for RNA extraction.

### RNA extraction and quantitative PCR

The total RNA of GFP-positive NIH 3T3 cells was extracted by RNAiso Plus (TaKaRa, Japan) according to the manufacturer's protocol and reverse-transcribed by ReverTra Ace® qPCR RT Master Mix with gDNA Remover (TOYOBO, Japan). Quantitative PCR reactions were performed with AceQ qPCR SYBR Green Master Mix (Vazyme, China) in QuantStudio 3 system (Thermo Fisher Scientific, USA). The primers used in this part are listed in Table S2.

### RNA sequencing analysis

The RNAs from GFP-positive NIH 3T3 cells were sent to HeQin Biotechnology Corporation (China) for library construction and RNA sequencing. Raw reads were processed using FASTP (V0.23.4) to filter out low-quality reads and remove sequencing adapters. Then, clean reads were aligned to the mouse genome (mm10) with STAR (V2.7.11a). Expression levels of tested genes were quantified by FeatureCounts (V2.0.3). Normalized expression value was calculated by the function normTransform from R package DESeq2 (V1.38.1) (Fig. S1B).

The off-target genes were predicted based on the sequence of crRNA. First, crRNA sequences were organized into FASTA format and input into the “seqkit locate” command to retrieve crRNA homologous sequences with up to five mismatches. Next, we converted the output of seqkit (V2.5.0) to BED format and then annotated it with homer (V4.11.1) to obtain genomic information of potential off-targets. Finally, a custom R script was employed to screen for potential off-targets located within exons. We obtained the corresponding gene names and conducted gene expression analysis to assess off-target effects.

### Animals

We purchased twelve-week-old male C57BL/6J mice from GemPharmatech (China). The male mice were housed in specific pathogen-free and individually ventilated cages. In animal rooms, the humidity and temperature were controlled at 55%–65% and 23 °C–27 °C respectively. Lights were turned on at 8 a.m. every morning and turned off at 8 p.m. Sufficient water and food were provided. We divided the whole male mice into five groups: sham with saline injection, DMM surgery with AAV5 (expressing EGFP), DMM surgery with AAV5 (expressing CasRx-C3C7), DMM surgery with AAV5 (expressing CasRx-S9S10), and DMM surgery with AAV5 (expressing CasRx-S9C7). DMM surgery was conducted as previously described.^32^ Ten days after DMM surgery, we conducted an intra-articular injection of 8 μL AAV5 for three months of treatment.

### Micro-CT analysis

We collected the right knee joints of sham and osteoarthritic mice that were injected with AAV5. We fixed the keen joints in 10% formalin for 48 h before scanning. We employed a NEMO Micro-CT scanner (Pingsheng Healthcare Shanghai Inc, China). Option (90 kV source and 70 μA current) were set to scan the samples with a resolution of 10 μm. We set the same thresholds to process all the scanned images from each group, allowing a 3-dimensional structural rendering of each sample.

### Histological analysis

The L4 dorsal root ganglia was fixed in 10% formalin for 24 h, followed by dehydration in 25% sucrose solution at 4 °C. The samples were then embedded in OCT (Sakura, Japan) at −20 °C. Serial coronal sections (12 μm) of dorsal root ganglia were cut using a cryostat microtome (Leica CM1950, Germany) and immediately subjected to immunofluorescence staining. The dorsal root ganglia sections were cleaned in phosphate buffer saline for 30 min and then sequentially treated with 10% bovine serum albumin and Triton X-100 at room temperature for 30 min (Beyotime, China). Next, the sections were incubated with primary antibodies diluted in 1% bovine serum albumin at 4 °C for 12 h. After cleaned in phosphate buffer saline for 45 min, the sections were incubated with a second antibody at room temperature for 120 min. Finally, the sections were cleaned in phosphate buffer saline for 45 min and mounted using VECTASHIELD Mounting Medium with DAPI (Vector Laboratories, USA). Images of immunofluorescence assays were captured by an Olympus VS120 microscope (Olympus VS120, Japan).

The right keen joints of mice were decalcified by 14% EDTA and then embedded in paraffin. Serial coronal sections of knee joints were cut every 4 μm from the medial compartments. The sections were performed safranin O/Fast-Green staining and Osteoarthritis Research Society International (OARSI) scoring as previously described.^33^ The damaged articular cartilage areas of knee joints were quantified by tracing the loss of safranin-O positive staining.

For immunohistochemistry staining, the sections of keen joints were sequentially incubated with 3% H_2_O_2_ (20 min), Triton X-100 (20 min), and 10% bovine serum albumin (60 min) after antigen retrieval. The operation of incubating the primary antibody and corresponding secondary antibody was the same as that of immunofluorescence staining. Finally, treatment with the VECTASTAIN Elite ABC Kit revealed immunohistochemistry signals by ImmPACT DAB Peroxidase Substrate (Vector Laboratories, USA). Images of immunohistochemistry staining were captured by a Zeiss Axioscan microscope (Zeiss, German). The quantification of all images in this step was employed using the Image J program. The antibodies used in this project are listed in Table S3.

### Statistical analysis

All the data were expressed as mean ± standard error of the mean, as shown in the figure legends. GraphPad Prism 9.0 was employed to conduct statistical analysis. Unpaired student's *t*-test (for two groups) and one-way ANOVA or two-way ANOVA (for multiple groups) followed by the Dunnett test, the Bonferroni test, or the Newman–Keuls test were used. *P* values < 0.05 were considered statistically significant.

## Results

### Efficient knockdown of the *Ctnnb1* or *Smo* transcripts using CasRx *in vitro*

**Figure 1:**
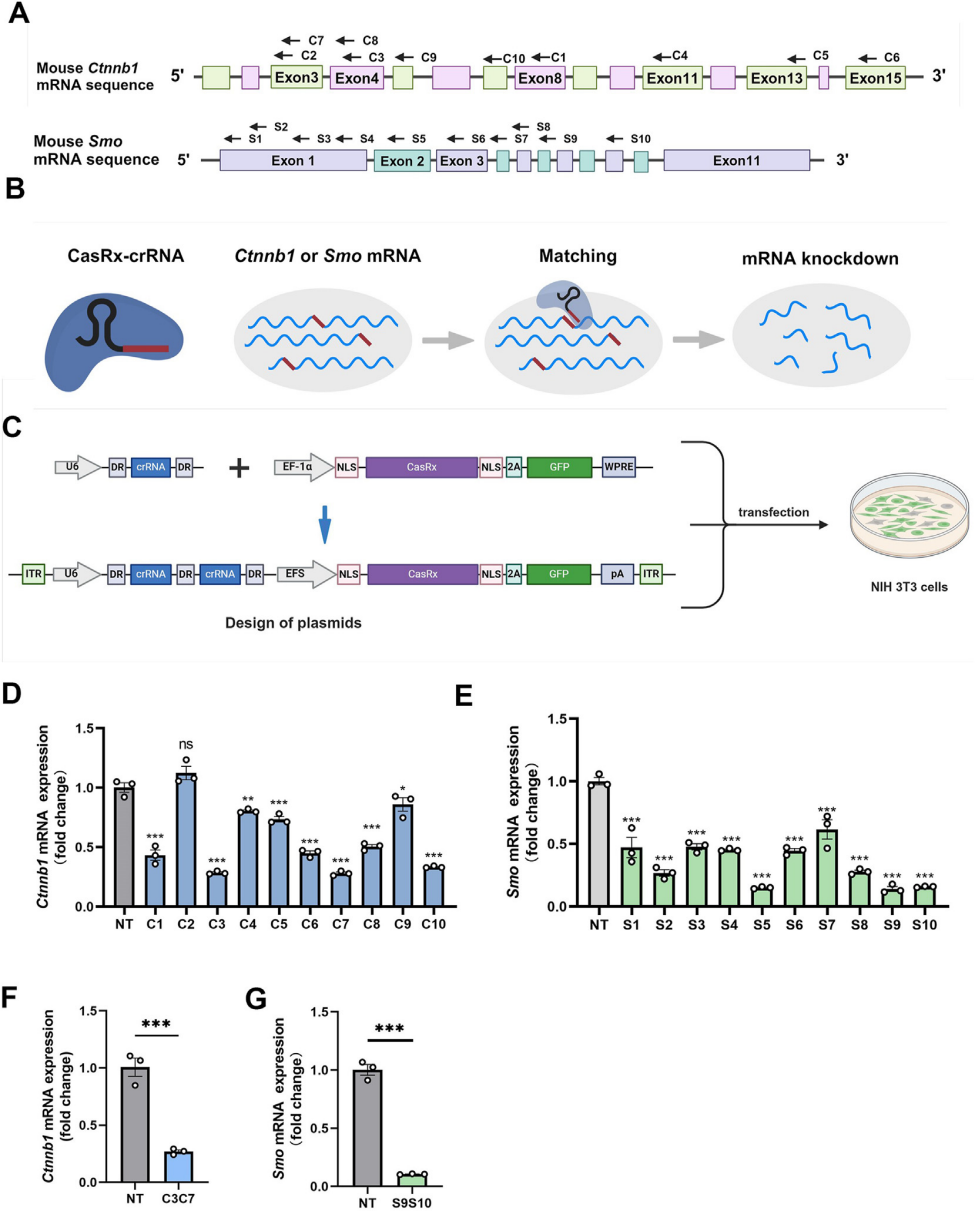
Efficient knockdown of the *Ctnnb1* or *Smo* transcripts using CasRx *in vitro*. **(A)** Design of 10 crRNAs matching *Ctnnb1* or *Smo* transcripts. **(B)** Schematic of *Ctnnb1* or *Smo* knockdown by CRISPR/CasRx. The sequences of the crRNA spacer are complementary to the *Ctnnb1* or *Smo* transcripts (bold red line). **(C)** Schematic of plasmids used for crRNA screening (upper) and crRNA combination (lower) of *Ctnnb1* or *Smo* knockdown in NIH 3T3 cells. Green cells indicated successful transfection of CasRx plasmids. **(D)** Knockdown of *Ctnnb1* by different crRNAs (C1 and C2) in NIH 3T3 cells. *n* = 3; one-way ANOVA followed by the Dunnett test. **(E)** Knockdown of *Smo* by different crRNAs (S1–S10) in NIH 3T3 cells. *n* = 3; one-way ANOVA followed by the Dunnett test. **(F)** Knockdown of *Ctnnb1* by combining C3 and C7 in NIH 3T3 cells. *n* = 3; unpaired student's *t*-test. **(G)** Knockdown of *Smo* by combining S9 and S10 in NIH 3T3 cells. *n* = 3; unpaired student's *t*-test. Data are represented as mean ± standard error of the mean; ∗*p* < 0.05, ∗∗*p* < 0.01, ∗∗∗*p* < 0.001.

We aimed to disrupt *Ctnnb1* and *Smo* mRNA efficiently in NIH 3T3 cells. We designed 20 crRNAs that match *Ctnnb1* (C1–C10) or *Smo* (S1–S10) transcripts, and a crRNA targeting *LacZ* was used as a non-targeting control (Fig. 1A, B). The sequences of all the crRNAs are listed in Table S4. We compared their knockdown efficiency by co-transfecting U6-pre-crRNA and CasRx-GFP into NIH 3T3 cells. 48 h after transfection, we sorted GFP-positive cells by fluorescence-activated cell sorting and performed quantitative PCR analysis (Fig. 1C). Our results demonstrated that all our designed crRNAs, except C2, revealed successful down-regulation of targeted mRNA. Among them, C3, C7, S9, and S10 showed the best effects, with the efficiency reaching 71.5% ± 0.8%, 72.0% ± 1.1%, 86.0% ± 1.9%, and 84.2% ± 0.3%, respectively (Fig. 1D, E). To ensure the efficiency of knockdown, we applied C3, C7, S9, and S10 combination treatments. We constructed two vectors that co-express C3C7 (or S9S10) and transfected them into NIH 3T3 cells, respectively (Fig. 1C). C3C7 and S910 caused a dramatic decrease in *Ctnnb1* and *Smo* expression, with the efficiency reaching 73.1% ± 1.5% and 89.5% ± 0.2%, respectively (Fig. 1F, G), with no difference compared with single crRNA knockdown. Taken together, these results indicated that CasRx could efficiently knock down *Ctnnb1* or *Smo* transcripts *in vitro*.

### No obvious off-target effects of CasRx-mediated *Ctnnb1* or *Smo* knockdown *in vitro*

**Figure 2:**
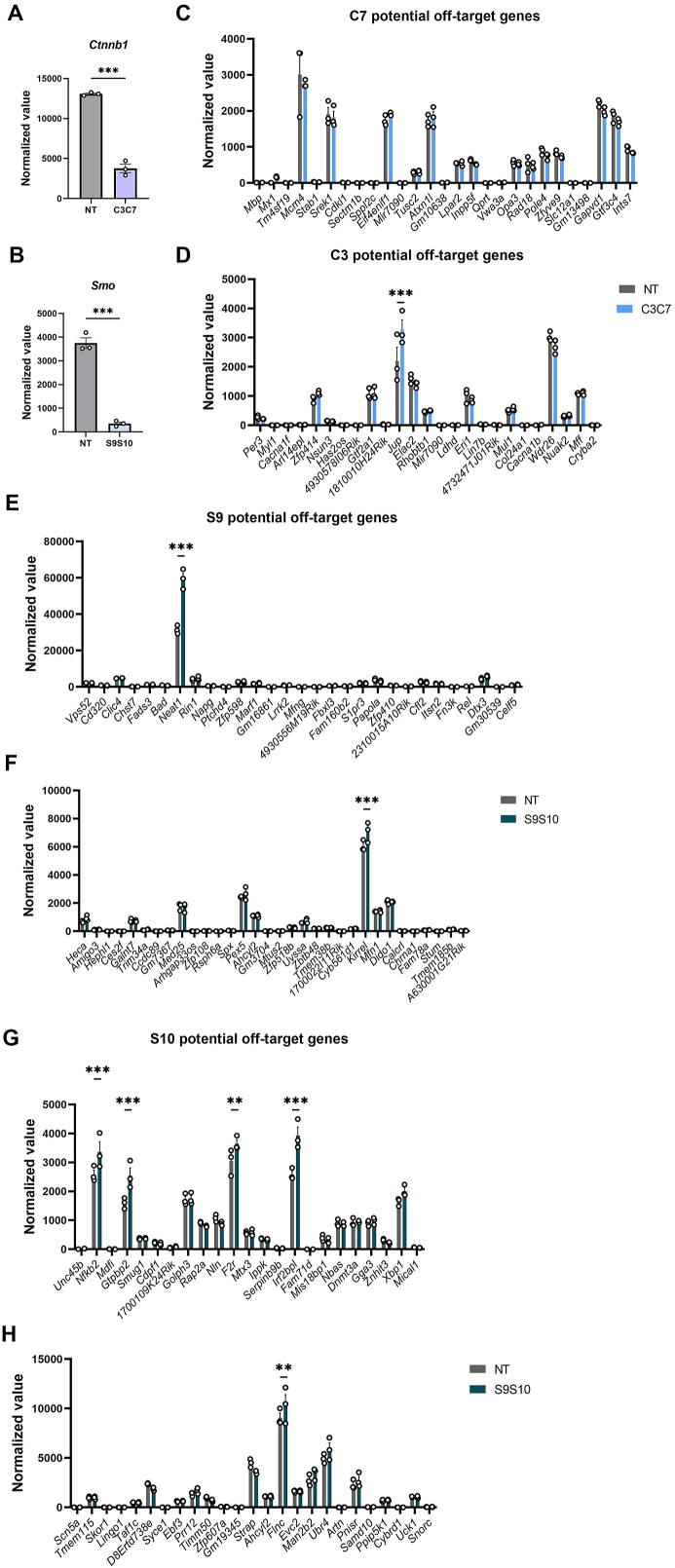
There were no obvious off-target effects of CasRx-mediated *Ctnnb1* or *Smo* knockdown *in vitro*. **(A)** Expression levels of *Ctnnb1* in the non-targeting (NT) and C3C7 group. *n* = 3; unpaired student's *t*-test. **(B)** Expression levels of *Smo* in the NT and S9S10 groups. *n* = 3; unpaired student's *t*-test. **(C, D)** Expression levels of C7-dependent (C) or C3-dependent (D) off-target genes were identified in the NT and C3C7 groups. *n* = 3; two-way ANOVA followed by the Bonferroni test. **(E**–**H)** Expression levels of S9-dependent (E, F) or S10-dependent (G, H) off-target genes were identified in the NT and S9S10 groups. *n* = 3; two-way ANOVA followed by the Bonferroni test. Data are represented as mean ± standard error of the mean; ∗*p* < 0.05, ∗∗*p* < 0.01, ∗∗∗*p* < 0.001.

To thoroughly detect the off-target effects of CasRx, we performed transcriptome-wide RNA sequencing of GFP-positive NIH 3T3 cells 48 h after transfection with CasRx-C3C7 or CasRx-S9S10 (Fig. 1C). As expected, CasRx caused a striking decrease of *Ctnnb1* or *Smo* transcripts (Fig. 2A, B). These results were consistent with the quantitative PCR data (Fig. 1F, G). Off-targets of the crRNAs were predicted based on their sequences. Up to five mismatch sites were set to search potential off-target genes. Among these potential off-targets, none of them were found with a significant decrease of expression in CasRx-C3C7 or CasRx-S9S10 transfected cells, when compared with non-targeting control (Fig. 2C–H). These results suggested that CasRx-mediated *Ctnnb1* or *Smo* mRNA knockdown has no obvious crRNA-dependent off-target effects in NIH 3T3 cells. Therefore, we chose C3C7 or S9S10 for *in vivo* gene therapy.

### Knockdown of *Ctnnb1* or *Smo* cannot ameliorate OA progression by CasRx

We prepared two AAV plasmids for *Ctnnb1* or *Smo* knockdown by CasRx *in vivo*. We chose AAV5 to deliver crRNA and CasRx because it can drive a long-lasting gene expression in the mouse knee joints.^4^^,^^34^ Before *in vivo* administration of the AAVs, we performed DMM surgery in mice. Ten days after DMM surgery, we performed intra-articular injections of AAV5 delivering CasRx-C3C7 or CasRx-S9S10 for treatment groups (Fig. 4A). In the non-treatment group, AAV5 expressing GFP was injected into mice. We evaluated joint tissues three months after the AAV5 injection, using histology and μCT analyses. The AAV5-CasRx expressed human influenza hemagglutinin (Fig. 4A). Immunofluorescence data showed hemagglutinin expression in the knee joints of the treatment group that were collected 3 months after injections of AAV5-CasRx (Fig. S2I). All the DMM groups showed evident joint degeneration (Fig. S2A). The μCT results showed obvious osteophyte formation in the keen joints (Fig. S2B). But it showed no significant difference in Osteoarthritis Research Society International (OARSI) score (Fig. S2C), synovitis score (Fig. S2D), osteophyte size (Fig. S2E), osteophyte maturity (Fig. S2F), and damaged area of cartilage (Fig. S2G) between the treatment groups and the control group when we carried out histological analysis to assess knee joint damage. Collectively, our data showed that CasRx-mediated *Ctnnb1* or *Smo* knockdown could not ameliorate OA progression, although *Smo* knockdown showed up-regulated expression in *Gli1* and *Patch1* mRNA in NIH 3T3 cells (Fig. S1A).

**Figure 3:**
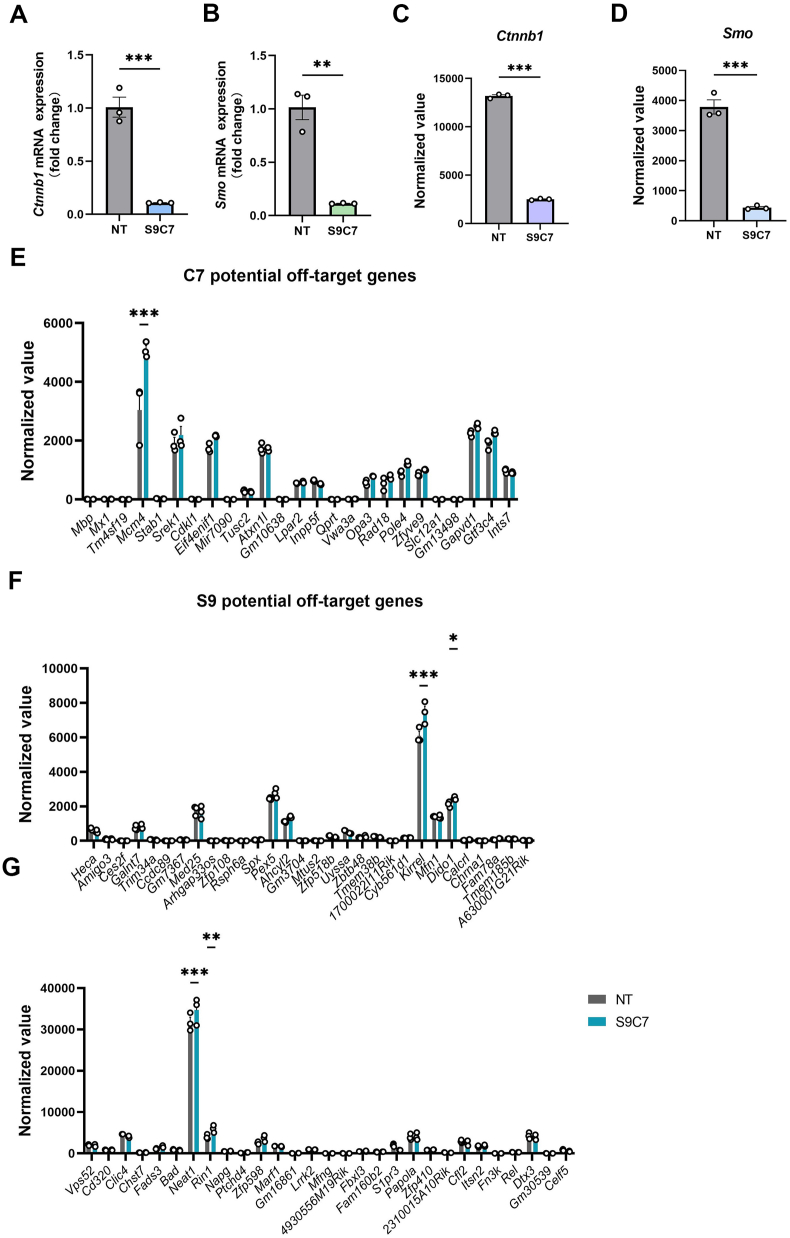
Efficient and specific knockdown of the *Ctnnb1* and *Smo* transcripts using CasRx *in vitro*. **(A, B)** Knockdown of *Ctnnb1* (A) and *Smo* (B) by combining S9 and C7 in NIH 3T3 cells. *n* = 3; unpaired student's *t*-test. **(C, D)***Ctnnb1* (C) and *Smo* (D) expression levels in the non-targeting (NT) and S9C7 group. *n* = 3; unpaired student's *t*-test. **(E**–**G)** Expression levels of C7-dependent (E) or S9-dependent (G, H) off-target genes were identified in the NT and S9C7 groups. *n* = 3; two-way ANOVA followed by the Bonferroni test. Data are represented as mean ± standard error of the mean; ∗*p* < 0.05, ∗∗*p* < 0.01, ∗∗∗*p* < 0.001.

**Figure 4:**
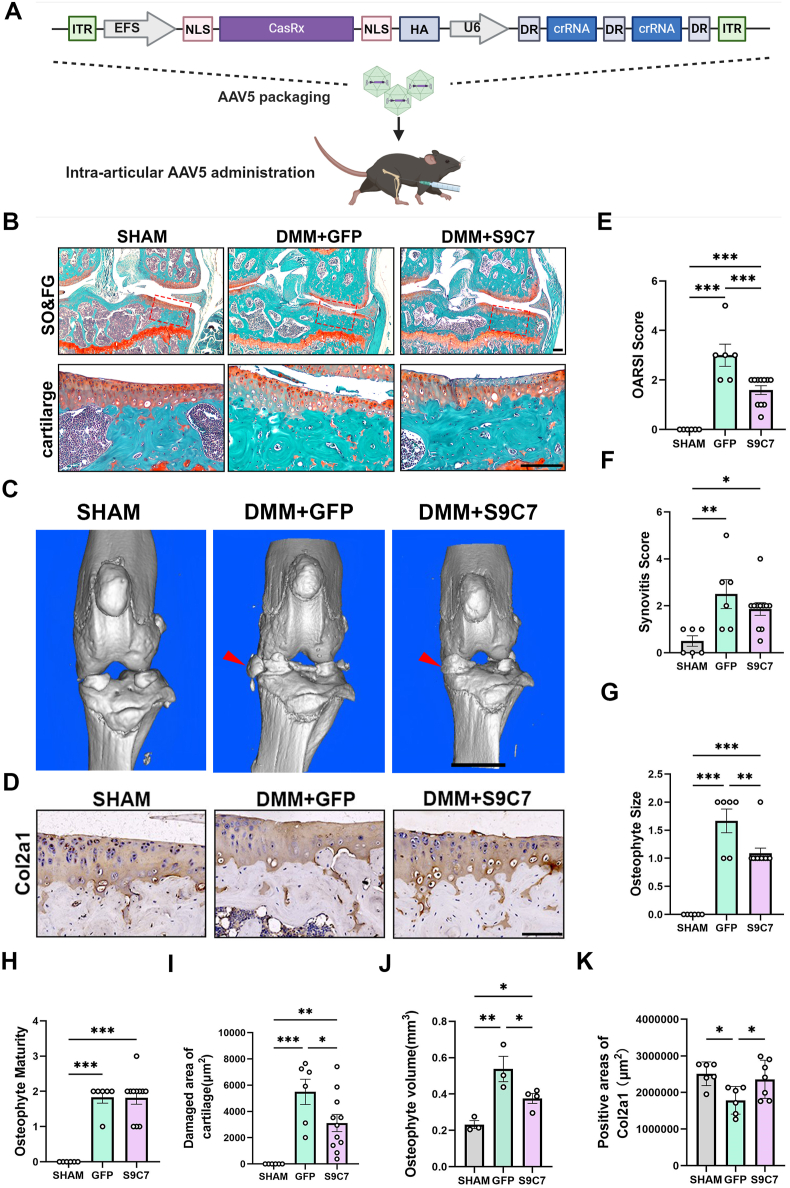
CasRx-mediated knockdown of *Ctnnb1* and *Smo* prevented OA keen joint degeneration. **(A)** Construction of AAV5 package plasmid. AAV5 delivered CasRx and S9C7 into the keen joint of DMM-induced OA mice. **(B)** Representative histology images of sham and osteoarthritic knee joints collected 3 months after injections of AAV5. Scale bar, 100 μm. *n* = 6–11. **(C)** Representative μCT images of sham and osteoarthritic knee joints collected 3 months after injections of AAV5. Red arrowheads, osteophytes. Scale bar, 2 mm. *n* = 3 or 4. **(D)** Representative Col2a1 immunohistochemistry images of sham and osteoarthritic knee joints collected 3 months after injections of AAV5. Scale bar, 100 μm. *n* = 6–11. **(E**–**H)** OARSI score (E), synovitis score (F), osteophyte size (G), and osteophyte maturity (H) of sham and osteoarthritic knee joints of the mice receiving AAVs. *n* = 6–11; one-way ANOVA followed by the Newman–Keuls test. **(I)** The damaged articular cartilage areas of knee joints were quantified by tracing the loss of safranin-O positive staining areas using the Image J system. *n* = 6–11; one-way ANOVA followed by the Newman–Keuls test. **(J)** Osteophyte volume of knee joints was quantified by μCT analysis in sham and osteoarthritic mice. *n* = 3 or 4; one-way ANOVA followed by the Newman–Keuls test. **(K)** Positive areas of Col2a1 of knee joints were quantified in sham and osteoarthritic mice. *n* = 6 or 7; one-way ANOVA followed by the Newman–Keuls test. Data are represented as mean ± standard error of the mean; ∗*p* < 0.05, ∗∗*p* < 0.01, ∗∗∗*p* < 0.001.

### Efficient and specific knockdown of the *Ctnnb1* and *Smo* transcripts using CasRx *in vitro*

To further explore CasRx-based gene knockdown as therapy for OA, we try to knock down *Ctnnb1* and *Smo* simultaneously. Based on previous data (Fig. 1D, E), we packaged S9, C7, and CasRx-GFP into one vector and tested its knockdown efficiency in NIH 3T3 cells. 48 h after CasRx-S9C7 transfection, the reduction of *Ctnnb1* and *Smo* mRNA successfully reached 89.2% ± 0.2% and 88.7% ± 0.2%, respectively (Fig. 3A, B). Next, we analyzed S9 and C7 sequence-dependent off-target effects by RNA sequencing. The CasRx caused a striking reduction of *Ctnnb1* and *Smo* mRNA (Fig. 3C, D) and no evident decrease in all the potential off-target genes (Fig. 3E–G). These data demonstrated that CasRx achieved efficient and specific knockdown of *Ctnnb1* and *Smo* simultaneously.

### CasRx-mediated knockdown of *Ctnnb1* and *Smo* prevents OA keen joint damage

To determine whether *Ctnnb1*-and *Smo*-targeting could preserve knee joints from OA damage in mice, we analyzed changes in joint tissues three months after receiving AAVs carrying CasRx-S9C7 (Fig. 4A). We employed safranin O and Fast-Green staining to examine whether CasRx-S9C7 prevents cartilage damages in OA mice (Fig. 4B). The articular cartilage revealed serious defects proved by significantly increased OARSI score in DMM surgery groups (Fig. 4E). We also quantified the damaged areas of articular cartilage by tracing the areas of cartilage loss by safranin O staining (Fig. 4I). The administration of CasRx-S9C7 led to a significant reduction in cartilage defects compared with the non-treatment group (Fig. 4E, I). The osteophyte size of the treatment group was significantly decreased compared with the non-treatment group (Fig. 4G), while no significant difference was found in osteophyte maturity between the treatment group and the non-treatment group (Fig. 4H). To further investigate the CasRx-S9C7 effect on osteophyte formation, we performed μCT analysis and found that osteophyte formation was markedly inhibited in the treatment group (Fig. 4G, J). In addition, there was no significant difference in synovitis score between the treatment group and the non-treatment group (Fig. 4F). We found that CasRx knockdown up-regulated expression of Col2a1 in articular cartilage of the treatment group (Fig. 4D, K). Our findings indicated that the knockdown of both *Ctnnb1* and *Smo* by CasRx significantly mitigated joint structure degeneration associated with OA progression.

### CasRx-mediated gene knockdown decreases OA-associated molecules in tissues

**Figure 5:**
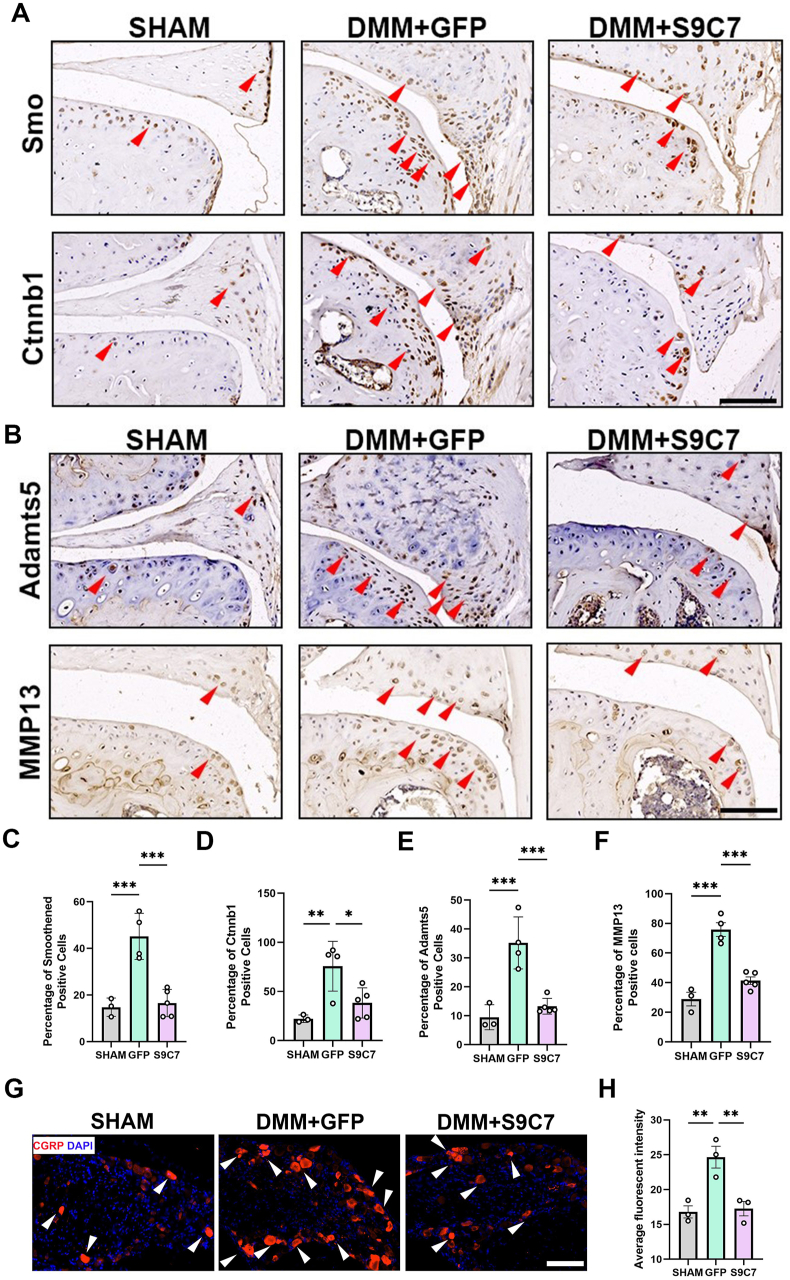
CasRx-mediated gene knockdown decreased OA-associated factors in tissues. **(A, B)** Representative Smo/Ctnnb1 (A) and Adamts5/MMP13 (B) immunohistochemistry images of sham and osteoarthritic knee joints collected 3 months after injections of AAV5. Red arrowheads, immunohistochemistry-positive cells. Scale bar, 100 μm. *n* = 3–5. **(C–F)** Percentage of Smo (C), Ctnnb1 (D), Adamts5 (E), and MMP13 (F) positive cells were quantified in knee joints of sham and osteoarthritic mice. *n* = 3–5; one-way ANOVA followed by the Newman–Keuls test. **(G, H)** Representative immunofluorescence images showed CGRP expression (G) and average fluorescence intensity of CGRP (H) in L4 dorsal root ganglia of sham and DMM mice collected 3 months after injections of AAV5. Scale bar, 100 μm. *n* = 3; one-way ANOVA followed by the Newman–Keuls test. Data are represented as mean ± standard error of the mean; ∗*p* < 0.05, ∗∗*p* < 0.01, ∗∗∗*p* < 0.001.

Next, we performed an immunohistochemistry analysis to investigate whether CasRx administration affected OA-associated gene expression. We examined the protein levels of Ctnnb1 and Smo and found that administration of CasRx-S9C7 decreased their expression compared with the GFP administration group in OA mice (Fig. 5A, C-D). These results demonstrated that CasRx could efficiently knock down *Ctnnb1* or *Smo* transcripts *in vivo*. We also analyzed the effects of CasRx-S9C7 treatment on catabolic genes. MMP13 and Adamts5 are two main matrix-degrading enzymes responsible for OA progression.^35^ The immunohistochemistry results showed that Adamts5 and MMP13 were significantly inhibited by CasRx-S9C7 treatment (Fig. 5B, E-F). It has been reported that calcitonin gene-related peptide (CGRP) expression increases in both the sensory nerve fibers of the periosteum and the dorsal root ganglia in OA mice.^38^ We observed CGRP up-regulation in DMM-induced OA mice. Treatment with CasRx-S9C7 significantly inhibited CGRP expression in L4 dorsal root ganglia tissues (Fig. 5G, H). However, it did not alleviate pain symptoms in OA mice (Fig. S3). Collectively, in this study, we ascertain that intra-articular RNA editing targeting both *Ctnnb1* and *Smo* exerts its positive influence on diverse joint tissues, including articular cartilage and menisci, thereby modulating downstream signaling pathways and changing the course of OA progression.

## Discussion

In this study, we applied CRISPR/CasRx-based RNA editing technique to treat OA, a chronic joint disease affecting multiple joint tissues in a DMM-induced mouse model. Our RNA editing study has indicated that targeting *Ctnnb1* and *Smo* transcripts simultaneously is a promising strategy for OA therapy, as our results showed a significant modification in articular cartilage damage and osteophyte formation when these molecules were down-regulated. Lorecivivint (SM04690), a β-catenin inhibitor, has revealed the potential to mitigate OA progression.^36^ It has undergone phase I (NCT02095548) and phase II (NCT02536833 and NCT03122860) clinical trials.^37^^,^^38^ It has been reported that interrupting the Ihh pathway, whether via inhibitors or gene modification, reduces the severity of OA in cartilage tissues of mice and humans *in vitro*.^39^, ^40^, ^41^, ^42^ However, our findings demonstrated that CasRx-mediated *Ctnnb1* or *Smo* knockdown showed no significant beneficial effects on a DMM-induced OA mouse model. Deleting exon 3 of β-catenin results in the production of a truncated β-catenin protein preventing GSK-3β mediated β-catenin phosphorylation and degradation.^25^ It is known that post-translation regulation, such as phosphorylation-mediated ubiquitin-proteasome degradation is the key regulatory mechanism for the functions of β-catenin and the Gli proteins, the key molecules for Ihh signaling. In contrast, transcriptional regulation of these molecules may not be critical enough to affect β-catenin and Ihh signaling. Our current findings support this notion. Nonetheless, the fact that the combination knockdown approach works well suggests that β-catenin and Ihh signaling molecules are potential therapeutic targets for OA treatment.

We reported intra-articular administration of the CRISPR/Cas9 system for deleting OA-related genes. We achieved positive effects of OA treatment,^4^ but irreversible genetic alterations seem overly hazardous for a condition like OA. CRISPR activation and CRISPR interference can manipulate gene transcription without genomic change.^43^ Recently, CRISPR activation/interference-mediated mesenchymal stromal cell engineering for OA treatment could be a promising approach. However, the above tools have a common disadvantage, that is, their sizes are too large to be delivered into the body. In this study, we chose the target genes whose activation leads to OA development and progression as therapeutic targets. The CRISPR/Cas13 technique provides a flexible approach to inhibit gene expression.^44^ It revealed more competent knockdown efficiency than traditional RNAi technology. CasRx is a type VI-D effector with 966 aa. Thus, it can be easily packaged into AAV and reveals tremendous potential for gene therapy.^14^ Because Cas13d can process its own CRISPR array, we can simultaneously deliver multiple targeting guides in a single-construction system for multiple gene knockdown.^14^ Off-target effects are a major concern for siRNA, a traditional RNA down-regulation tool.^45^ In this study, we did not observe obvious crRNA-dependent off-target effects by RNA sequencing analysis. Performing intra-articular instead of systemic administration further minimizes the unwanted side effects. A proper delivery tool is also crucial. Although AAV5 can drive long-lasting gene expression in murine joints,^4^^,^^34^ it still cannot achieve satisfactory efficiency. In our study, the knockdown of *Ctnnb1* or *Smo* cannot alleviate OA progression. It may also be due to the low infection efficiency of AAV5. Using the combination targeting approach may be able to enhance the effect and overcome the low infection efficiency of AAV5. The more potent delivery vectors targeting articular cartilage need to be developed in the future.

In conclusion, gene knockdown with the CRISPR/CasRx technique inhibits Wnt/β-catenin and Ihh signaling and alleviates OA progression in mice. This approach may have a therapeutic potential for OA in humans.

## Ethics declaration

All the animal protocols complied with the guidelines of the Ethics Committee of the Shenzhen Institute of Advanced Technology, Chinese Academy of Sciences (SIAT-IACUC-220211-YYS-CD-A2114).

## CRediT authorship contribution statement

**Xingyun Huang:** Writing – original draft, Formal analysis, Data curation. **Jiamin Yu:** Formal analysis, Data curation. **Shixue Gou:** Formal analysis, Data curation. **Hongyu Qin:** Formal analysis, Data curation. **William W. Lu:** Writing – review & editing, Supervision, Project administration, Funding acquisition. **Zhen Li:** Writing – review & editing. **Liping Tong:** Writing – review & editing. **Di Chen:** Writing – original draft, Supervision, Project administration, Funding acquisition, Conceptualization.

## Conflict of interests

The authors declared no competing interests.

## Funding

This project was supported by the National Key Research and Development Program of China (No. 2022YFA1207500), the National Natural Science Foundation of China (No. 82394445, 82250710174, 82161160342, 82030067 to D.C.), the Research Grants Council (RGC) of Hong Kong, China (No. HKU-17101821 to W.W.L. and D.C.), and the Shenzhen Science and Technology Research Funding (China) (No. JSGGKQTD20210831174330015 to D.C.).

## Data availability

RNA sequencing data have been deposited with links to BioProject accession number PRJNA1141616 in the NCBI BioProject database (https://www.ncbi.nlm.nih.gov/bioproject/).
