# Supplementary material for: CRISPR/CasRx-mediated RNA knockdown targeting β-catenin and Ihh signaling alleviates osteoarthritis
Source: Genes Dis. 2024 Nov 16;12(4):101468. doi: 10.1016/j.gendis.2024.101468 (PMC12033902; doi:10.1016/j.gendis.2024.101468)
Supplement: Multimedia component 1 [file mmc1.docx]

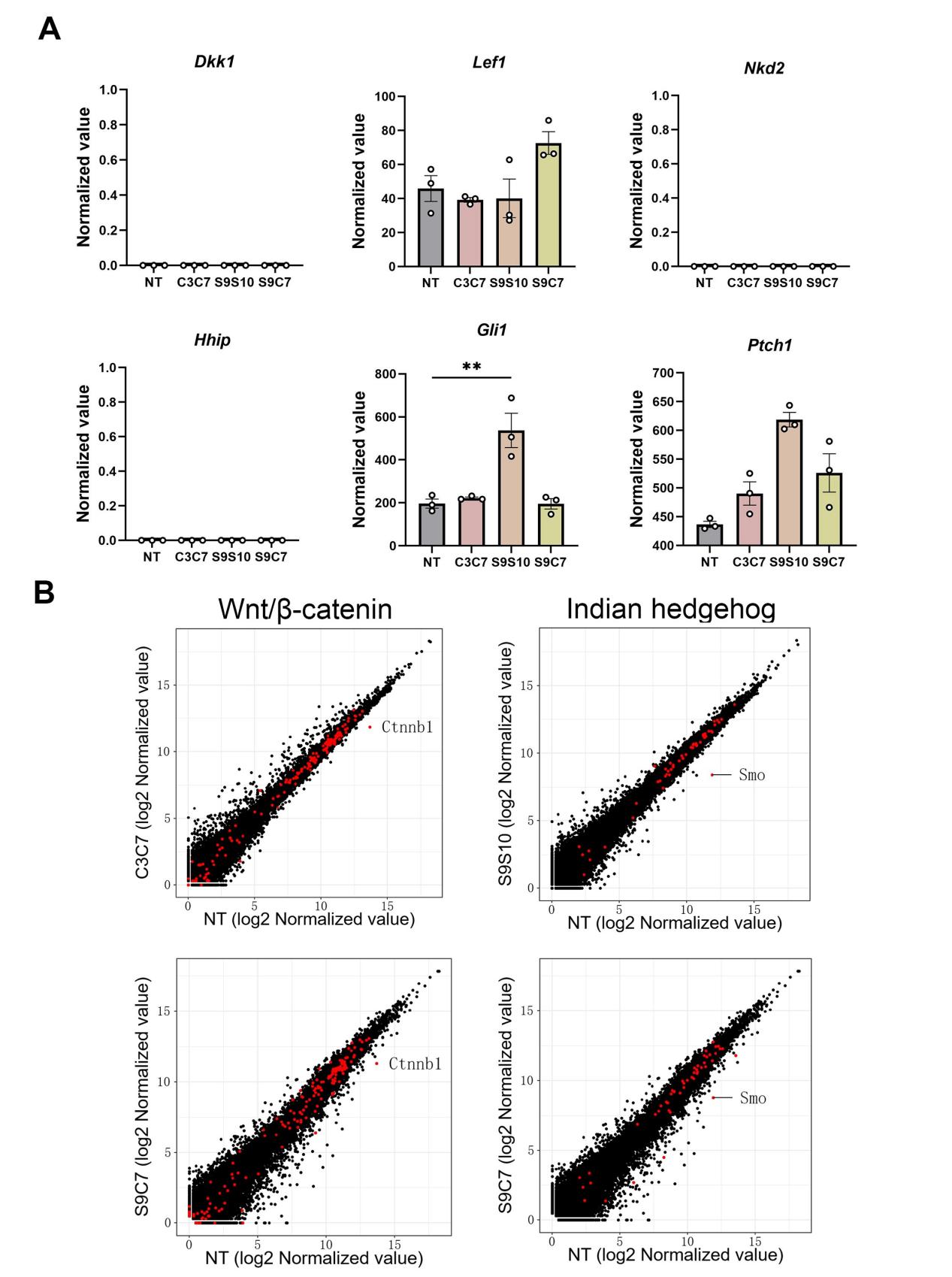


**Figure S1**. (A) The expression of Wnt/β-catenin (*Dkk1*, *Lef1*, *Nkd2*) and/or Indian hedgehog signaling target genes (*Hhip*, *Gli1*, *Ptch1*). n=3. One-way ANOVA followed by the Dunnett test. (B) The expression of genes in Wnt/β-catenin signaling pathway and Indian hedgehog signaling pathways at different experimental conditions. Genes in Wnt/β-catenin signaling pathway or Indian hedgehog signaling pathway were highlighted in red color. Normalized expression value was calculated by the function normTransform from R package DESeq2 (V1.38.1). Data are represented as mean ± SEM. * *p*<0.05, ** *p*<0.01, *** *p*<0.001.


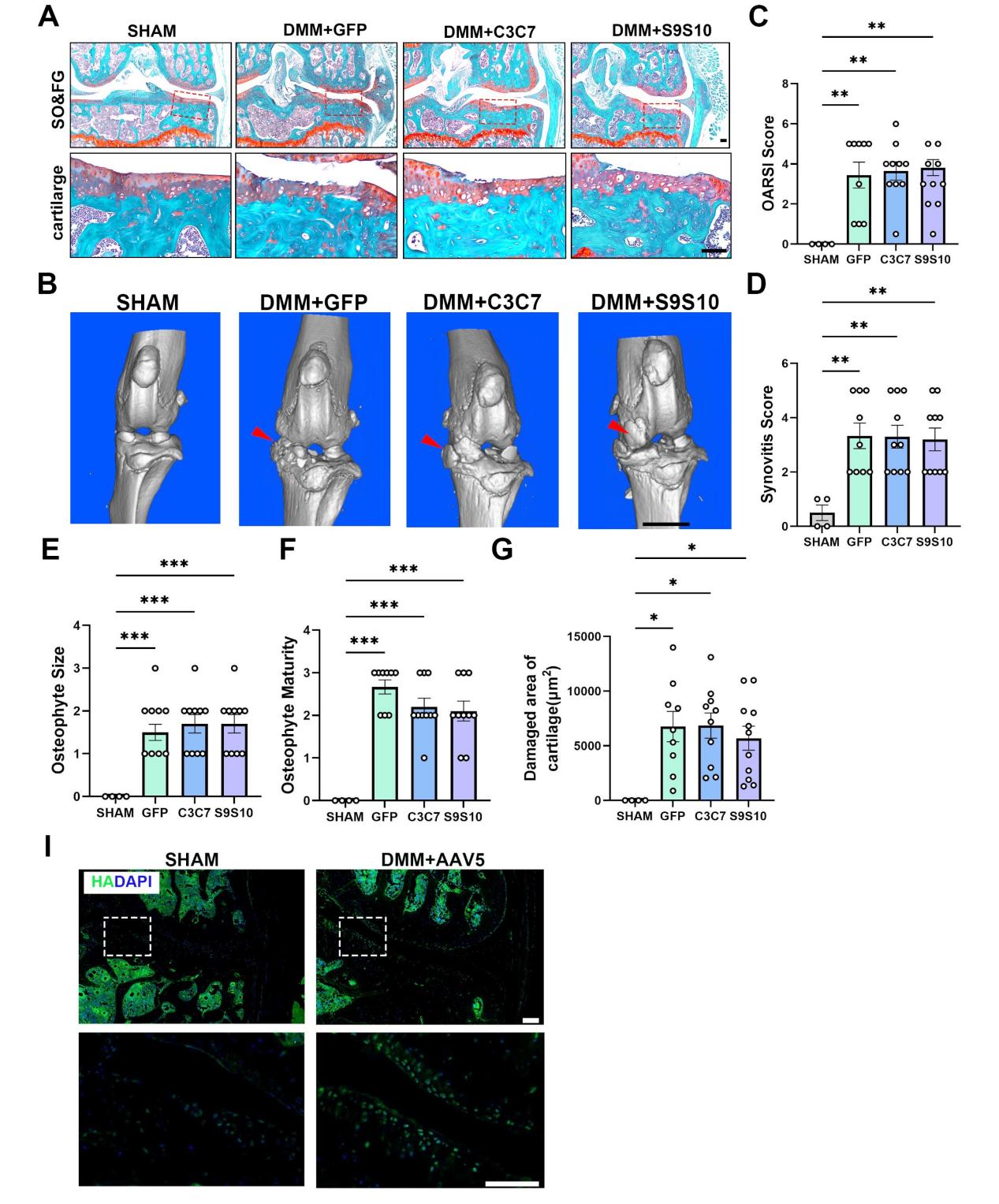


**Figure S2.** Knockdown of *Ctnnb1* or *Smo* cannot ameliorate OA progression by CasRx (A) Representative histology images of sham and osteoarthritic knee joints that were collected 3 months after injections of AAV5. Scale bar: 100 µm. n=4-10. (B) Representative d μCT images of sham and osteoarthritic knee joints that were collected 3 months after injections of AAV5. Red arrowheads, osteophytes. Scale bar: 2 mm. n=4-10. (C-F) OARSI score (C), synovitis score (D), osteophyte size (E), and osteophyte maturity (F) of sham and osteoarthritic knee joints of the mice receiving AAVs. n=4-10. One-way ANOVA followed by the Newman-Keuls test. (G) The damaged articular cartilage areas of knee joints were quantified by tracing the loss of Safranin-O positive stained areas using the Image J system. n=4-10. One-way ANOVA followed by the Newman-Keuls test. Data are represented as mean with SEM, **p*<0.05, ***p*<0.01, ****p*<0.001. (I) Representative IF images showed HA tag expression in articular cartilage of Sham and DMM mice that were collected 3 months after injections of AAV5-CasRx. Scale bar: 100 µm. n=3.


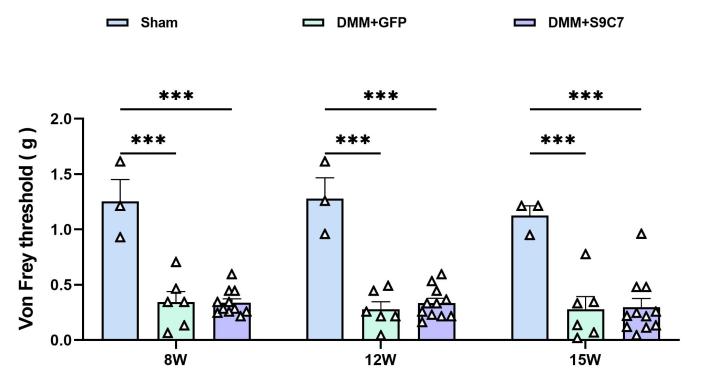


**Figure S3**. The von Frey test showing the sensitivity to mechanical allodynia in Sham or DMM mice with AAV5-GFP or AAV5-S9C7 treatment as indicated time points. n=3-11. Two-way ANOVA followed by the Bonferroni test. Data are represented as mean with SEM, **p*<0.05, ***p*<0.01, ****p*<0.001.

Table S1. Primer sequences used for construction plasmids

| Primers | Sequences（5’to 3’） |
| --- | --- |
| mSmo-crRNA1-F | AAACtcccaccattaaaaccgactccc |
| mSmo-crRNA1-R | CTTGgggagtcggttttaatggtggga |
| mSmo-crRNA2-F | AAACgcagcaactgcagcagcctccggggcgcca |
| mSmo-crRNA2-R | CTTGtggcgccccggaggctgctgcagttgctgc |
| mSmo-crRNA3-F | AAACggctggtcaccggcacgtccctc |
| mSmo-crRNA3-R | CTTGgagggacgtgccggtgaccagcc |
| mSmo-crRNA4-F | AAACttccggaggccggaccagagcacgagcttg |
| mSmo-crRNA4-R | CTTGcaagctcgtgctctggtccggcctccggaa |
| mSmo-crRNA5-F | AAACggaagtggtccggtgtgcaacgc |
| mSmo-crRNA5-R | CTTGgcgttgcacaccggaccacttcc |
| mSmo-crRNA6-F | AAACgcctcggtgaacagcgggttctgacactga |
| mSmo-crRNA6-R | CTTGtcagtgtcagaacccgctgttcaccgaggc |
| mSmo-crRNA7-F | AAACcggcgggcaccatccatgaactg |
| mSmo-crRNA7-R | CTTGcagttcatggatggtgcccgccg |
| mSmo-crRNA8-F | AAACgccaccgtgaggacaaaggggag |
| mSmo-crRNA8-R | CTTGctcccctttgtcctcacggtggc |
| mSmo-crRNA9-F | AAACgccttctcactcagaagcccagg |
| mSmo-crRNA9-R | CTTGcctgggcttctgagtgagaaggc |
| mSmo-crRNA10-F | AAACctgcagcagctcacgccgcttag |
| mSmo-crRNA10-R | CTTGctaagcggcgtgagctgctgcag |
| mCtnnb1-crRNA1-F | AAACccttcctgcttagtcgctgcatc |
| mCtnnb1-crRNA1-R | CTTGgatgcagcgactaagcaggaagg |
| mCtnnb1-crRNA2-F | AAACtcagctacttgctcttgcgtgaaggactgg |
| mCtnnb1-crRNA2-R | CTTGccagtccttcacgcaagagcaagtagctga |
| mCtnnb1-crRNA3-F | AAACcaacatctgtgatggttcagcca |
| mCtnnb1-crRNA3-R | CTTGtggctgaaccatcacagatgttg |
| mCtnnb1-crRNA4-F | AAACgagcaaggatgtggagagctccagtacacc |
| mCtnnb1-crRNA4-R | CTTGggtgtactggagctctccacatccttgctc |
| mCtnnb1-crRNA5-F | AAACgcagtctcattccaagccattggctctgtc |
| mCtnnb1-crRNA5-R | CTTGgacagagccaatggcttggaatgagactgc |
| mCtnnb1-crRNA6-F | AAACcccatccatgaggtcctgggcgt |
| mCtnnb1-crRNA6-R | CTTGacgcccaggacctcatggatggg |
| mCtnnb1-crRNA7-F | AAACggtggcaccagaatggattccag |
| mCtnnb1-crRNA7-R | CTTGctggaatccattctggtgccacc |
| mCtnnb1-crRNA8-F | AAACgcgtctcagggaacatggcagct |
| mCtnnb1-crRNA8-R | CTTGagctgccatgttccctgagacgc |
| mCtnnb1-crRNA9-F | AAACcccaagcattttcaccagcgctg |
| mCtnnb1-crRNA9-R | CTTGcagcgctggtgaaaatgcttggg |
| mCtnnb1-crRNA10-F | AAACctctgcttgtggtccacagaagc |
| mCtnnb1-crRNA10-R | CTTGgcttctgtggaccacaagcagag |
| LacZ-crRNA-F | AAACcgtctggccttcctgtagccagctttcatc |
| LacZ-crRNA-R | CTTGgatgaaagctggctacaggaaggccagacg |
| C3C7-R  C3C7-F | agtcgaggcatcgatgcttgatatcgaattcttacttgtacag  gttccagattacgctgctagcggcagtggagag |

Table S2. Primers used for qPCR

| Primers | Sequences（5’ to 3’） |
| --- | --- |
| Q-mCtnnb1-F1  Q-mCtnnb1-R1 | ATGGAGCCGGACAGAAAAGC  CTTGCCACTCAGGGAAGGA |
| Q-mGapdh-F1  Q-mGapdh-R1 | AGGTCGGTGTGAACGGATTTG  TGTAGACCATGTAGTTGAGGTCA |
| Q-mSmo-F1  Q-mSmo-R1 | GAGCGTAGCTTCCGGGACTA  CTGGGCCGATTCTTGATCTCA |

Table S3. Antibody used in this paper

| Antibodies | Catalog number | Source | Concentrations |
| --- | --- | --- | --- |
| Anti-Smo polyclonal antibody | 20787-1-AP | Proteintech | 1:300 |
| anti-Ctnnb1 polyclonal antibody | ET1601-5 | HUABIO | 1:500 |
| anti-Adamts5 polyclonal antibody | 31158 | Signal way antibody | 1:100 |
| anti-MMP13 polyclonal antibody | 18165-1-AP | Proteintech | 1:300 |
| Anti-Collagen II antibody | ab34712 | Abcam | 1:200 |
| Anti-HA tag antibody | ab305269 | Abcam | 1:500 |
| Goat Anti-Rabbit IgG (H&L)-HRP Conjugated | BE0101 | EASYBIO | 1:200 |
| Goat Anti-Mouse IgG (H&L)-HRP Conjugated | BE0102 | EASYBIO | 1:200 |
| anti-CGRP polyclonal antibody | C7113 | Sigma | 1:300 |
| Cy5-conjugated AffiniPure Goat Anti-Mouse IgG H&L | HY-P81018 | MCE | 1:200 |

Table S4. Sequences information of crRNA

| Genes | Name and sequences（5’-3’） |
| --- | --- |
| *Smo* (NM_176996) | S1: tcccaccattaaaaccgactccc |
|  | S2: gcagcaactgcagcagcctccggggcgcca |
|  | S3: ggctggtcaccggcacgtccctc |
|  | S4: ttccggaggccggaccagagcacgagcttg |
|  | S5: ggaagtggtccggtgtgcaacgc |
|  | S6: gcctcggtgaacagcgggttctgacactga |
|  | S7: cggcgggcaccatccatgaactg |
|  | S8: gccaccgtgaggacaaaggggag |
|  | S9: gccttctcactcagaagcccagg |
|  | S10: ctgcagcagctcacgccgcttag |
| *Ctnnb1* (NM_007614) | C1: ccttcctgcttagtcgctgcatc |
|  | C2: tcagctacttgctcttgcgtgaaggactgg |
|  | C3: caacatctgtgatggttcagcca |
|  | C4: gagcaaggatgtggagagctccagtacacc |
|  | C5: gcagtctcattccaagccattggctctgtc |
|  | C6: cccatccatgaggtcctgggcgt |
|  | C7: ggtggcaccagaatggattccag |
|  | C8: gcgtctcagggaacatggcagct |
|  | C9: cccaagcattttcaccagcgctg |
|  | C10: ctctgcttgtggtccacagaagc |
| *LacZ* | NT: cgtctggccttcctgtagccagctttcatc |
